# Provider‐ and patient‐level predictors of oral anticancer agent initiation and adherence in patients with metastatic renal cell carcinoma

**DOI:** 10.1002/cam4.4201

**Published:** 2021-09-04

**Authors:** Lisa P. Spees, Stephanie B. Wheeler, Bradford E. Jackson, Christopher D. Baggett, Lauren E. Wilson, Melissa A. Greiner, Deborah R. Kaye, Tian Zhang, Daniel George, Charles D. Scales, Jessica E. Pritchard, Michael Leapman, Cary P. Gross, Michaela A. Dinan

**Affiliations:** ^1^ Department of Health Policy and Management Gillings School of Global Public Health University of North Carolina at Chapel Hill (UNC‐CH) Chapel Hill North Carolina USA; ^2^ Lineberger Comprehensive Cancer Center (LCCC) UNC‐CH Chapel Hill North Carolina USA; ^3^ Department of Epidemiology Gillings School of Global Public Health UNC‐CH Chapel Hill North Carolina USA; ^4^ Department of Population Health Sciences Duke University School of Medicine (DUSM) Durham North Carolina USA; ^5^ Duke Cancer Institute (DCI) Center for Prostate and Urologic Cancers Durham North Carolina USA; ^6^ Department of Medicine DUSM Durham North Carolina USA; ^7^ Department of Surgery (Urology) DUSM Durham North Carolina USA; ^8^ Department of Urology Yale School of Medicine New Haven Connecticut USA; ^9^ Cancer Outcomes, Public Policy, and Effectiveness Research (COPPER) Center Yale School of Medicine New Haven Connecticut USA; ^10^ Department of Medicine Yale School of Medicine New Haven Connecticut USA; ^11^ Department of Chronic Disease Epidemiology Yale School of Public Health New Haven Connecticut USA

**Keywords:** adherence, initiation, metastatic, oral anticancer agents, renal cell carcinoma

## Abstract

**Background:**

Improving oral anticancer agent (OAA) initiation and adherence is the important quality‐of‐care issues, particularly since one fourth of anticancer agents being developed will be administered orally. Our objective was to identify provider‐ and patient‐level characteristics associated with OAA initiation and adherence among individuals with metastatic renal cell carcinoma (mRCC).

**Methods:**

We used state cancer registry data linked to multi‐payer claims data to identify patients with mRCC diagnosed in 2004–2015. Provider data were obtained from North Carolina Health Professions Data System and the National Plan & Provider Enumeration System. We estimated risk ratios (RRs) and corresponding 95% confidence limits (CLs) using modified Poisson regression to evaluate factors associated with OAA initiation and adherence.

**Results:**

Among the 207 (out of 687) patients who initiated an OAA following mRCC diagnosis and survived 90 days, median proportion of days covered was 0.91. Patients with a modal provider specializing in hematology/medical oncology were much more likely to initiate OAAs than those seen by other specialties. Additionally, patients with a female provider were more likely to initiate OAAs than those with a male provider. Compared to patients treated by providers practicing in both urban and rural areas, patients with providers practicing solely in urban areas were more likely to initiate OAAs, after controlling for patient‐level factors (RR = 1.37; 95% CL: 1.09–1.73). Medicare patients were less likely to be adherent than those with private insurance (RR = 0.61; 95% CL: 0.42–0.87).

**Conclusions:**

Our results suggest that provider‐ and patient‐level factors influence OAA initiation in patients with mRCC but only insurance type was associated with adherence.

## Graphical abstract

While several patient‐level factors have been associated with oral anticancer agent (OAA) initiation and adherence for metastatic renal cell carcinoma (mRCC) and other cancers, few provider‐level factors have been examined, despite providers being a key component driving OAA access. We examined provider and patient characteristics associated with OAA initiation and adherence among individuals with mRCC. Our results suggest that provider‐ and patient‐level factors are associated with OAA initiation but only patient‐level factors are associated with adherence.

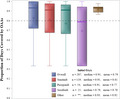

## 1 INTRODUCTION

Renal cell carcinoma (RCC) leads to more years of life lost than any other genitourinary cancer in the United States.^1^, ^2^ An estimated 73,750 new cases of RCC are diagnosed annually, and approximately 558,000 patients currently live with the disease. Although survival is excellent for patients with localized RCC, only 13% of those diagnosed with metastatic disease will survive 5 years.^2^ Since 2005, the US Food and Drug Administration (FDA) have approved several first‐ and second‐line oral anticancer agents (OAAs) to treat metastatic renal cell carcinoma (mRCC).^3^ These include first‐ and second‐generation tyrosine kinase inhibitors whose pharmacokinetics require oral administration and offer convenience relative to infused therapies. Given that one fourth of anticancer agents being developed will be administered orally,^4^, ^5^ improving OAA initiation and adherence has become an important quality‐of‐care issue.

Despite increased use, few published studies have examined the predictors of OAA initiation and adherence among mRCC patients in real‐world settings.^6^, ^7^, ^8^, ^9^, ^10^ While several patient‐level factors such as socioeconomic status, race/ethnicity, age, and comorbidities have been associated with OAA initiation and adherence for mRCC and other cancers, few provider‐level factors have not been examined. Providers not only control access to OAAs but also influence OAA adherence; previous work suggests that providers’ knowledge of OAAs as well as their attitudes and support of OAAs influence adherence.^11^, ^12^ To optimize the outcomes in mRCC patients, multiple levels of influence within the healthcare system, including provider factors, need to be taken into account, and a better understanding of the larger context in which medication‐taking barriers should be addressed is needed.^13^, ^14^, ^15^ Our study examined both provider‐ and patient‐level factors associated with initiation of and adherence to OAAs in a real‐world cohort of individuals newly diagnosed with mRCC.

## 2 METHODS

### 2.1 Study population

We conducted a retrospective cohort study of patients diagnosed with mRCC and their providers between 2004 and 2015. Patient data were obtained from the University of North Carolina Cancer Information Population Health Resource, a resource that links the North Carolina Central Cancer Registry data to administrative claims data from private health insurance, Medicare, and Medicaid plans across the state.^16^ Provider data were obtained from North Carolina Health Professions Data System and the National Plan & Provider Enumeration System.

**FIGURE 1:**
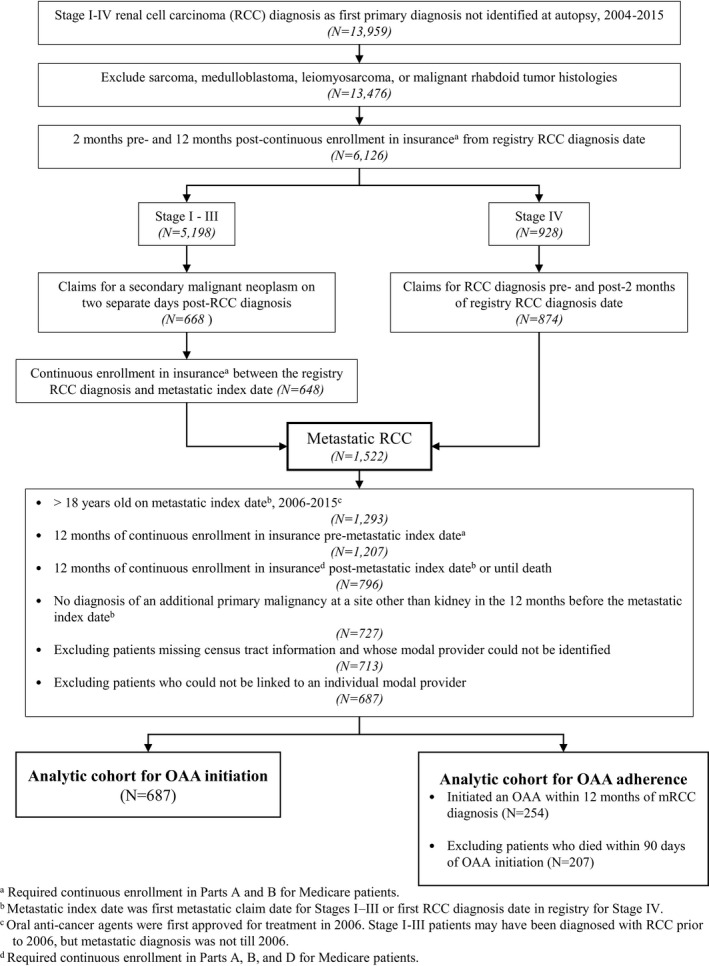
Metastatic Renal cell carcinoma (RCC) Cohort Algorithm

Figure 1 illustrates how we arrived at our analytic cohorts. In brief, eligible patients included those initially diagnosed with stage I–IV RCC. Stage I–III patients were included if they had claims for secondary malignant neoplasm on two separate days at any time after their registry recorded initial RCC diagnosis; stage IV patients were included if they had claims‐identifiable codes for RCC within 2 months of their registry recorded diagnosis date. The index date was defined as the RCC diagnosis date from the registry for stage IV patients, or the date of the first of the two metastatic diagnoses claims for stage I–III patients.

We defined the modal provider as the individual who was identified most frequently on claims with a diagnosis code of RCC or metastatic cancer between 2 months prior to and 3 months following the index date. Individual providers were identified using their National Provider Identifier code.

We defined two cohorts of patients. The first cohort included all patients with mRCC (*N* = 687), and our outcome of interest was OAA initiation. For the second cohort, we only included patients with mRCC from the first cohort who had initiated an OAA and survived the first 90 days post‐initiation (*N* = 207). For this cohort, the outcome of interest was OAA adherence. Throughout the manuscript, the first cohort will be referred to as the mRCC cohort and the second cohort will be referred to as the OAA initiator cohort.

### 2.2 Study variables

Oral anticancer agent initiation within the 12 months following the patient's metastatic index date was identified from prescription drug files and pharmacy claims by reviewing generic and brand names as well as national drug codes for the following: sorafenib (2005), sunitinib (2006), pazopanib (2009), everolimus (2009), and axitinib (2012). Adherence to OAAs was defined as having ≥80% proportion of days covered (PDC) for the 90 consecutive days following an initial OAA claim that patients had access to any OAA days’ supply. For sunitinib claims, the days’ supply was adjusted to account for the “4 week on, 2 week off” scheduling, to avoid underestimating adherence.^7^, ^8^, ^17^

Provider‐level variables included specialty, sex, race/ethnicity, years in practice, provider's RCC patient volume, and practice locations. Provider specialty was classified as urology/urological surgery, hematology/medical oncology, internal medicine, and other. Provider's years in practice was calculated for each provider–patient encounter as the years elapsed from the provider's medical school graduation to the year of the patient's index date. Provider volume was defined as the frequency of unique RCC patients that the provider treated prior to an mRCC patient's diagnosis date.

Patient‐level control variables of interest included: age at metastatic diagnosis, race/ethnicity, rural location (based on the revised 2010 Rural Urban Commuting Area code categorization^18^), patient sex, insurance coverage at metastatic index date, histology, stage at initial diagnosis, radical/partial nephrectomy in the prior year, number of comorbidities at baseline (based on comorbidities included in the Charlson Comorbidity Index^19^), the predicted probability of frailty,^20^ and distance to the nearest National Cancer Institute Cancer Center. In addition, patients’ area‐level socioeconomic context was measured using 2008–2012 American Community Survey 5‐year estimates of census tract‐level percent with bachelor's degree and percent living below the poverty level.

### 2.3 Statistical analysis

We estimated the frequencies, percentages, medians, and interquartile ranges (IQR) of provider‐ and patient‐level characteristics for both the mRCC and OAA initiator cohorts. Poisson regression with a robust variance estimator was used to estimate the unadjusted and multivariable adjusted risk ratios (RRs) and their corresponding 95% confidence limits (CLs) to evaluate factors associated with OAA initiation and adherence. As a sensitivity analysis, we used a fractional logit model to evaluate the unadjusted and adjusted PDC.

For both the OAA initiation and adherence analyses, a model reduction method was chosen to account for the analytic sample sizes, the number of study variables of interest, and recommended events per variable (EPV) guidelines. We calculated the maximum allowable number of variables based on at least 10 EPV and used a least absolute shrinkage and selection operator (LASSO) approach to minimize bias in the selection of covariates in the multivariable models from the list of aforementioned provider and patient variables.^21^ Consequently, for both outcomes, all provider‐level covariates, including specialty, sex, race/ethnicity, years in practice, RCC patient volume, and practice location were selected for inclusion. When the outcome of interest was OAA initiation, patient‐level variables selected for inclusion in multivariate analyses included age, sex, location, insurance, number of comorbid conditions, census tract‐level percent with bachelor's degree and percent living below the poverty level, nephrectomy in 12 months prior, stage at diagnosis, distance to the nearest NCI‐designated center, and frailty. When the outcome of interest was OAA adherence, patient‐level variables included in the adjusted model selected using the LASSO approach were age, sex, race/ethnicity, location, insurance, number of comorbid conditions, census tract‐level percent living below the poverty level, nephrectomy in 12 months prior, and distance to the nearest NCI‐designated center. Multicollinearity of covariates was assessed using a variance inflation factor of 5. *p*‐values <0.05 were considered to be statistically significant. Analyses were conducted using SAS v9.4 (Cary, NC).

## 3 RESULTS

### 3.1 Analytic samples

Of the 687 patients in the mRCC cohort, 37% initiated an OAA in the 12 months following metastatic diagnosis. The median age at diagnosis was 70 years, and the majority were male (66%), NH White (77%), and urban‐residing (62%) (Table 1). Most were covered by Medicare only at the time of metastatic diagnosis (63%). A third of patients had three or more comorbid conditions (33%). With respect to provider characteristics, most modal providers were with hematologists/medical oncologists (56%), males (83%), and NH white (74%), and had a median of 20 years in practice. Compared with the full mRCC cohort, the initiator cohort (*N *= 207) was younger, had fewer comorbidities, was more often male, and privately insured.

**TABLE 1:** Descriptive statistics of the analytic cohorts

	mRCC cohort (*n* = 687)		OAA initiator cohort (*n* = 207)	
	*n*/Median	%/IQR	*n*/Median	%/IQR
Patient characteristics				
Age at diagnosis (years)				
Median, IQR	69.5	61.0, 76.3	67.0	57.3, 73.0
18–49	43	6.3	19	9.2
50–64	172	25.0	66	31.9
65–69	132	19.2	46	22.2
70–74	128	18.6	31	14.9
75–79	107	15.6	33	15.9
80+	105	15.3	12	5.8
Sex				
Female	236	34.4	62	30.0
Male	451	65.7	145	70.1
Race/ethnicity				
Non‐Hispanic White	527	76.7	164	79.2
Non‐Hispanic Black	125	18.2	32	15.5
Other^a^	35^a^	5.1	*	*
Location				
Urban	424	61.7	133	64.3
Rural	263	38.3	74	35.8
Insurance at diagnosis				
Private	134	19.5	61	29.5
Any Medicaid	123	17.9	35	16.9
Medicare only	430	62.6	111	53.6
Number of comorbid conditions				
0	137	19.9	59	28.5
1	193	28.1	66	31.9
2	134	19.5	38	18.4
3+	223	32.5	44	21.3
% Poverty^b^	17.1	10.6, 23.8	16.8	10.7, 24.3
% <BA degree^b^	83.1	70.1, 88.5	82.4	69.7, 88.6
Histology				
Clear cell	628	91.4	191	92.3
Other	59	8.6	16	7.7
Nephrectomy in 12 months prior				
No	546	79.5	160	77.3
Yes	141	20.5	47	22.7
Stage at diagnosis				
I	122	17.8	27	13.0
II	45	6.6	23	11.1
III	124	18.1	45	21.7
IV	396	57.6	112	54.1
Frailty^b^	0.05	0.03, 0.12	0.03	0.02, 0.08
Distance (miles) to nearest NCI‐cancer center^b^, ^c^	65.4	37.6, 91.10	63.8	35.5, 90.8
Provider characteristics (patient‐level**)**				
Specialization				
Hematology/medical oncology	386	56.2	151	73.0
Urology/urological surgery	165	24.0	47	22.7
Internal medicine	96	14.0	*	*
Other	40	5.8	*	*
Sex				
Male	567	82.5	174	84.1
Female	120	17.5	33	15.9
Race/ethnicity				
Non‐Hispanic White	506	73.7	150	72.5
Non‐Hispanic Black	44	6.4	13	6.3
Asian/Pacific Islander	95	13.8	30	14.5
Other	42	6.12	14	6.3
Years in practice^b^	20.0	13,28	20	13,28
Volume^b^, ^d^	10	4,32	9	2,23
Location				
Rural only	66	9.6	*	*
Urban only	456	66.4	157	75.9
Rural and urban	165	24.0	40	19.3

Abbreviations: BA, bachelor; IQR, interquartile range; mRCC, metastatic renal cell carcinoma; NCI, National Cancer Institute; OAA, oral anticancer agent.

^a^ “Other” patient race/ethnicity comprised: non‐Hispanic American Indian, non‐Hispanic Other, Hispanic White, Hispanic Other, Unknown ethnicity White, and Unknown ethnicity Black.

^b^ Median and IQR are shown.

^c^ NCI centers were included from states contiguous with North Carolina: Georgia, South Carolina, Tennessee, and Virginia.

^d^ Provider volume was defined as the frequency of unique RCC patients treated prior to a mRCC diagnosis.

* Indicates cell value is ≤11 and is suppressed to protect patients’ confidentiality.

### 3.2 OAA initiation among mRCC patients

In unadjusted and adjusted models, patients whose modal providers’ specialties were other than hematology/medical oncology were far less likely to initiate OAAs compared to patients treated by other specialties. Specifically, in the adjusted models, patients with modal providers specializing in urology/urological surgery (RR = 0.67; 95% CI: 0.53, 0.86), internal medicine (RR = 0.19; 95% CI: 0.10, 0.36), or other specialties (RR = 0.15; 95% CI: 0.05, 0.46) were less likely to initiate OAAs compared to patients with modal providers specializing in hematology/medical oncology. In the adjusted model only, patients with female modal providers (RR = 1.28; 95%CL: 1.00, 1.62) or providers that practiced in only urban areas (RR = 1.37; 95% CL: 1.09, 1.73) were more likely to initiate OAAs. Additionally, adjusted models indicated that patients ages 80+ years of age (RR = 0.39; 95% CL: 0.23, 0.66) and those with 3+ comorbid conditions (RR = 0.72; 95% CL: 0.55, 0.94) were less likely to initiate OAAs than, respectively, patients ages 18–49 years and those with no comorbid conditions (Table 2). Patients with greater predicted frailty were also less likely to initiate OAAs (RR = 0.78; 95% CL: 0.63, 0.95).

**TABLE 2:** Risk ratios (RRs) for OAA initiation among patients with mRCC (*N* = 687)

	Unadjusted RR (95% CL)	*p* ^a^	Adjusted RR (95% CL)	*p* ^a^
Patient characteristics				
Age at diagnosis				
18–49	Ref		Ref	
50–64	0.80 (0.58, 1.09)	0.16	0.84 (0.61, 1.14)	0.25
65–69	0.73 (0.52, 1.02)	0.07	0.81 (0.56, 1.19)	0.29
70–74	**0.55 (0.38, 0.80)**	**0.002**	0.68 (0.44, 1.03)	0.07
75–79	0.74 (0.52, 1.04)	0.09	0.97 (0.65, 1.43)	0.87
80+	**0.27 (0.16, 0.46)**	**<0.001**	**0.39 (0.23, 0.66)**	**<0.001**
Sex				
Female	Ref		Ref	
Male	1.19 (0.96, 1.48)	0.11	1.05 (0.86, 1.30)	0.61
Race/ethnicity				
Non‐Hispanic White	Ref		—	
Non‐Hispanic Black	0.90 (0.69, 1.18)	0.45		
Other	0.99 (0.63, 1.54)	0.96		
Patient location				
Urban	Ref		Ref	
Rural	0.96 (0.78, 1.17)	0.66	1.09 (0.89, 1.34)	0.39
Insurance at diagnosis				
Private	Ref		Ref	
Any Medicaid	**0.73 (0.55, 0.97)**	**0.03**	1.14 (0.84, 1.54)	0.40
Medicare only	**0.63 (0.51, 0.78)**	**<0.001**	0.93 (0.69, 1.26)	0.65
Number of comorbid conditions				
0	Ref		Ref	
1	0.83 (0.65, 1.05)	0.12	0.88 (0.69, 1.11)	0.27
2	**0.66 (0.49, 0.88)**	**0.01**	0.79 (0.59, 1.04)	0.10
3+	**0.56 (0.43, 0.74)**	**<0.001**	**0.72 (0.55, 0.94)**	**0.02**
% Poverty				
Highest quartile versus lower	1.12 (0.9, 1.4)	0.29	1.21 (0.98, 1.49)	0.08
% <BA degree				
Highest quartile versus lower	1.16 (0.94, 1.44)	0.17	1.01 (0.82, 1.24)	0.95
Histology				
Clear cell	Ref		—	
Other	1.01 (0.72, 1.43)	0.95		
Nephrectomy in 12 months prior	0.94 (0.74, 1.21)	0.66	0.89 (0.67, 1.17)	0.39
Stage at diagnosis				
IV	Ref		Ref	
I	**0.59 (0.42, 0.83)**	**0.003**	**0.63 (0.46, 0.87)**	**0.005**
II	**1.37 (1.01, 1.85)**	**0.04**	1.29 (0.95, 1.77)	0.11
III	0.98 (0.76, 1.27)	0.89	0.96 (0.73, 1.27)	0.78
Distance to nearest NCI‐designated center				
Highest quartile versus lower	0.89 (0.70, 1.13)	0.33	0.94 (0.75, 1.17)	0.56
Frailty				
Above median versus below	**0.64 (0.52, 0.79)**	**<0.001**	**0.78 (0.63, 0.95)**	**0.02**
Provider characteristics				
Specialization				
Hematology/medical oncology	Ref		Ref	
Urology/urological surgery	**0.67 (0.53, 0.86)**	**0.002**	**0.59 (0.46, 0.77)**	**<0.001**
Internal medicine	**0.19 (0.10, 0.36)**	**<0.001**	**0.22 (0.12, 0.41)**	**<0.001**
Other	**0.15 (0.05, 0.46)**	**0.0008**	**0.19 (0.06, 0.55)**	**0.002**
Sex				
Male	Ref		Ref	
Female	1.1 (0.84, 1.44)	0.50	**1.28 (1.00, 1.62)**	**0.048**
Race/ethnicity				
Non‐Hispanic White	Ref		Ref	
Non‐Hispanic Black	1.07 (0.72, 1.57)	0.75	1.32 (0.90, 1.93)	0.15
Asian/Pacific Islander	1.07 (0.81, 1.42)	0.61	0.98 (0.77, 1.24)	0.84
Other	1.06 (0.70, 1.60)	0.78	1.17 (0.78, 1.76)	0.45
Years in practice^b^	1.00 (1.00, 1.00)	0.92	1.00 (1.00, 1.00)	0.66
Volume^c^	0.99 (0.98, 1.01)	0.26	0.99 (0.98, 1.01)	0.43
Location				
Rural only	0.62 (0.36, 1.06)	0.08	0.84 (0.49, 1.43)	0.52
Urban only	1.28 (1.00, 1.64)	0.05	**1.37 (1.09, 1.73)**	**0.007**
Rural and urban	Ref		Ref	

Abbreviations: BA, bachelor; CL, confidence limits; mRCC, metastatic renal cell carcinoma; NCI, National Cancer Institute; OAA, oral anticancer agent.

^a^ Bolded estimates have *p*‐values <0.05.

^b^ Years in practice was scaled to 5 years.

^c^ Provider volume was scaled to four RCC patients.

### 3.3 PDC among OAA initiators

**FIGURE 2:**
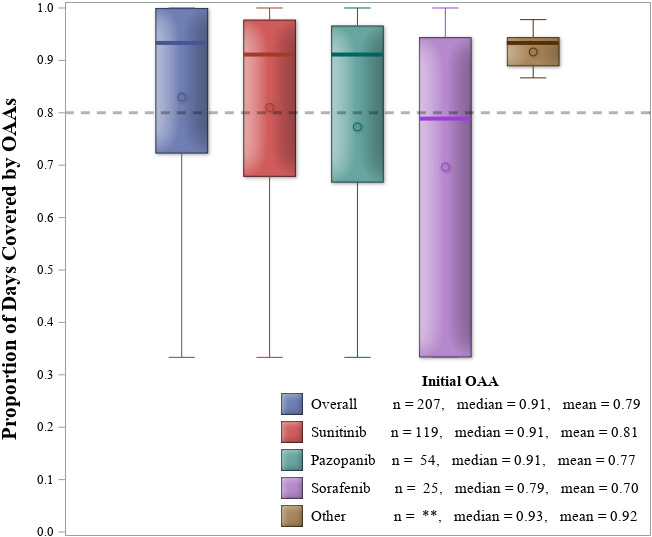
Distribution of proportion of days covered from first prescribed oral anticancer agent (OAA) over 90 days following initial OAA claim, stratified by select OAAs. The circles represent the mean and heavy vertical line represents the median. The box represents the interquartile range and whiskers represent the range of values. The category “Other” includes axitinib and everolimus. **indicates cell value is ≤11 and is suppressed to protect patients’ confidentiality

Among the 207 patients who initiated an OAA and survived at least 90 days after initiation, the median PDC was 0.91 (IQR: 0.66, 0.97) (Figure 2). The most frequently prescribed OAAs were sunitinib (57%) and pazopanib (26%). Adherence during the initial 90 days was similar for all OAAs except sorafenib (median = 0.79; IQR: 0.33, 0.94), which was lower (Figure 2; Table S1).

Table 3 presents the RRs for the ≥80% PDC adherence outcome. Multivariable analysis showed that those only insured by Medicare were less likely to be adherent (RR = 0.61; 95% CL: 0.42, 0.87) than those covered by private insurance. Sensitivity analyses demonstrated the mean PDC among privately insured patients was 0.89, which was significantly higher when compared to patients with Medicaid (ΔPDC = −0.13; 95% CL: −0.22, −0.04) and with Medicare only (ΔPDC = −0.14; 95% CL: −0.20, −0.08; Table S2).

**TABLE 3:** Risk ratios (RRs) for 80% OAA adherent among patients with mRCC who survived at least 90 days post‐OAA initiation (*N* = 207)

	Unadjusted RR (95% CL)	*p* ^a^	Adjusted RR (95% CL)	*p* ^a^
Age at diagnosis				
18–49	Ref		Ref	
50–64	0.94 (0.71, 1.23)	0.63	0.91 (0.70, 1.19)	0.49
65–69	**0.70 (0.50, 1.00)**	**0.049**	0.97 (0.64, 1.47)	0.89
70–74	0.82 (0.58, 1.16)	0.26	1.11 (0.71, 1.72)	0.64
70+	**0.65 (0.45, 0.94)**	**0.02**	0.87 (0.56, 1.36)	0.55
Sex				
Female	Ref		Ref	
Male	1.16 (0.91, 1.48)	0.23	1.09 (0.84, 1.40)	0.51
Race/ethnicity				
Non‐Hispanic White	Ref		—	
Other	0.83 (0.61, 1.11)	0.21	0.84 (0.63, 1.10)	0.22
Patient location				
Urban	Ref		Ref	
Rural	**0.77 (0.60, 0.98)**	**0.03**	0.86 (0.67, 1.10)	0.22
Insurance at diagnosis				
Private	Ref		Ref	
Any Medicaid	**0.67 (0.50, 0.91)**	**0.01**	0.72 (0.48, 1.06)	0.10
Medicare only	**0.64 (0.52, 0.78)**	**<0.001**	**0.61 (0.42, 0.87)**	**0.007**
Number of comorbid conditions				
0	Ref		Ref	
1	0.84 (0.61, 1.06)	0.12	0.87 (0.67, 1.13)	0.30
2	1.08 (0.84, 1.39)	0.56	1.22 (0.95, 1.57)	0.12
3+	0.85 (0.63, 1.15)	0.29	1.07 (0.79, 1.44)	0.66
% Poverty				
Highest quartile versus lower	0.85 (0.66, 1.11)	0.23	0.95 (0.75, 1.22)	0.70
% <BA degree				
Highest quartile versus lower	0.91 (0.71, 1.17)	0.47	—	
Histology				
Clear cell	Ref		—	
Other	1.08 (0.76, 1.53)	0.65		
Nephrectomy in 12 months prior	1.07 (0.85, 1.35)	0.56	1.07 (0.86, 1.33)	0.55
Stage at diagnosis				
IV	Ref		—	
I	0.94 (0.69, 1.30)	0.72		
II	0.85 (0.58, 1.24)	0.40		
III	0.92 (0.70, 1.20)	0.55		
Distance to nearest NCI‐designated center				
Highest quartile versus lower	1.09 (0.84, 1.4)	0.52	0.95 (0.74, 1.21)	0.67
Frailty				
Above median versus below	0.98 (0.79, 1.22)	0.87	—	
**Provider characteristics**				
Specialization				
Hematology/medical oncology	Ref		Ref	
Other^b^	1.16 (0.94, 1.43)	0.18	1.08 (0.87, 1.35)	0.46
Sex				
Male	Ref		Ref	
Female	0.78 (0.55, 1.10)	0.16	0.79 (0.57, 1.12)	0.18
Race/ethnicity				
Non‐Hispanic White	Ref		Ref	
Other^c^	0.79 (0.60, 1.03)	0.085	0.86 (0.66, 1.13)	0.29
Years in practice^d^	1.00 (1.00, 1.00)	0.71	1.00 (1.00, 1.00)	0.53
Volume^e^	1.00 (0.99, 1.01)	0.80	1.00 (0.99, 1.01)	0.98
Location				
Urban only	1.37 (1.02, 1.84)	0.04	1.34 (0.99, 1.81)	0.05
Rural and urban	Ref		Ref	

Abbreviations: BA, bachelor; CL, confidence limits; mRCC, metastatic renal cell carcinoma; OAA, oral anti‐cancer agent.

^a^ Bolded estimates have *p*‐values <0.05.

^b^ Due to small cell sizes, categories of urology/urological surgery, internal medicine, and other were collapsed into one category.

^c^ Due to small cell sizes, catergories of Non‐Hispanic Black, Asian/Pacific Islander, and other were collapsed into one category.

^d^ Years in practice was scaled to 5 years.

^e^ Provider volume was scaled to 4 RCC patients and covers the period between the patient’s metastatic index date and all prior years of data.

## 4 DISCUSSION

In this novel study, among patients with mRCC, OAA initiation was significantly associated with provider‐level factors, including specialization, sex, and practice location, after controlling for patient‐level factors previously shown to be meaningful predictors of OAA initiation in this patient population.^6^, ^7^, ^8^, ^9^, ^10^ Among those who initiated OAAs and survived at least 90 days post‐initiation, median PDC was high at 91%, suggesting substantial overall adherence among diverse patients with mRCC starting oral therapies. However, publicly insured patients, particularly those on Medicare, have lower adherence to OAAs over time, relative to privately insured patients. In contrast to OAA initiation, provider‐level variables were not associated with OAA adherence. Taken together, these findings highlight different barriers to the initiation and maintenance of OAAs.

Several provider‐level variables were positively associated with OAA initiation, including having a modal provider in a hematology/oncology specialty. Compared to other specialists, hematologists/oncologists may be better able to address some of the key barriers to initiating OAAs and feel more equipped to manage treatment‐related toxicities. Their increased familiarity and experience with OAAs may mean they are more comfortable and adept at educating and counseling patients on using OAAs than other providers. Second, the process of obtaining insurance authorization of OAAs is onerous and may represent an impediment to prescribing OAAs for providers who are not routinely managing cancer patients.^13^ For example, medical oncology practices are more likely to have clinical oncology pharmacists or other staff specially trained in facilitating access to OAAs.^22^ Not only does this reduce a potential process barrier to accessing OAAs, but strong care coordination through oncology and pharmacy providers has been associated with patients’ beliefs in the necessity of OAAs and the perceived benefits of taking OAAs, which may affect the long‐term adherence.^23^

Providers practicing in urban areas were also more likely to initiate OAAs. Significant differences in OAA initiation have been documented among cancer patients residing in rural and urban areas.^24^, ^25^, ^26^ These disparities may be partially attributed to the differences in resources and clinical volume between urban and rural practices. In a previous study among patients with prostate and kidney cancer receiving OAAs, 73% of all OAA prescriptions required two or more phone calls by clinic staff, and 40% required five or more calls.^27^ Providers practicing in urban clinics may have more staff they can rely on to ensure that patients’ prescriptions are authorized and completed than smaller, rural practices. Furthermore, providers in rural areas are more likely to be professionally isolated and face barriers to continuing medical education,^28^ potentially making them less aware of current guideline and best treatment practices.

Even though OAA adherence was high, PDC was 14 percentage points lower among Medicare patients than those with private insurance, even when normalized for age, frailty, and comorbid conditions. This disparity, in part, may be due to Medicare patients facing significant cost barriers. Compared to privately insured patients, Medicare patients generally have higher coinsurance costs for specialty drugs, and there is no cap on out‐of‐pocket spending for outpatient prescription drugs.^13^, ^29^ Consequently, as out‐of‐pocket spending from OAAs accumulates, the financial burden experienced by Medicare patients may reduce their OAA adherence over time. While 78% of OAA prescriptions filled with Medicare Part D receive some type of charitable assistance, these programs often only cover about 15% of the OAA prescription's cash price.^30^ If OAA prices continue to annually increase by 12%,^31^ these insurance disparities may further harm OAA adherence and potentially also influence OAA initiation, particularly in Medicare‐insured individuals.

There are limitations of this study that should be noted. First, our study only includes individuals from a single state, potentially limiting the generalizability of our findings. However, the present study from a racially, geographically, and socioeconomically diverse cohort confirms and reinforces findings from other claims‐based studies using MarketScan and SEER‐Medicare, showing that age and comorbidities are associated with OAA initiation.^9^, ^32^ Importantly, our study adds provider‐level predictors to the literature on mRCC OAA use. Second, while we were able to observe the provider visits for which a patient had insurance claims to identify the modal provider, we do not know the content of those visits and assumed, as other analyses have been performed, that the modal provider was most likely to have influence over patients’ OAA use. Lastly, OAA adherence was measured using filled prescription records rather than observing actual medication use; filled prescriptions do not necessarily indicate they were consumed, but this is a common approach in the literature that is used to understand medication use.^6^, ^8^

Using a population‐based, multi‐payer sample, our results suggest that provider characteristics, including specialty and location, are linked with OAA initiation, although more information is needed to better understand provider decision‐making, knowledge, and level of comfort prescribing and managing OAAs in this patient population. Finally, while OAA adherence was high overall, we found evidence of disparities in use among Medicare patients compared to privately insured patients. Future research should examine the role that insurance plays within the larger healthcare context and particularly how it interacts with OAA adherence over time.^13^

## ETHICS STATEMENT

This study received exemption status by the University of North Carolina Institutional Review Board (#19‐0451).

## CONFLICTS OF INTEREST

LPS and BEJ receive unrelated funding paid to their institution from AstraZeneca. SBW has received unrelated grant funding paid to her institution from Pfizer Foundation/NCCN and AstraZeneca. TZ has research funding and consulting relationships with Pfizer, Merck, and Novartis, and consulting relationships with Exelixis, Calithera, and Bayer. DG has a current relationship with Bayer, Novartis, Merck & Co., Exelixis, and Pfizer. CG has received research funding from the NCCN Foundation, Genentech, Johnson & Johnson, and funding from Flatiron Inc. for travel to and speaking at a scientific conference. CS has research funding with Pfizer, Exelixis, Merck, and BMS.

## Supporting information

Table S1–S2

## Data Availability

The individual registry and claims data (even de‐identified) used and/or analyzed during this study are not publicly available due to the Cancer Information and Population Health Resource (CIPHR) policies. Collaboration requests and data use agreements with CIPHR (https://ciphr.unc.edu/) are necessary to obtain access to the de‐identified data.
