# Supplementary material for: Provider‐ and patient‐level predictors of oral anticancer agent initiation and adherence in patients with metastatic renal cell carcinoma
Source: Cancer Med. 2021 Sep 4;10(19):6653–65. doi: 10.1002/cam4.4201 (PMC8495289; doi:10.1002/cam4.4201)
Supplement: Supplementary file 1 — Table S1–S2 [file CAM4-10-6653-s001.docx]

| **Supplemental Table 1.** Distribution of proportion of days covered from first prescribed OAA over 90 days following their initial drug claim, stratified by select OAAs | | | |
| --- | --- | --- | --- |
|  | **N** | **Mean (SD)** | **Median (IQR)** |
| Overall | 207 | 0.79 (0.23) | 0.91 (0.66, 0.97) |
| Sunitinib | 119 | 0.81 (0.21) | 0.91 (0.68, 0.98) |
| Pazopanib | 54 | 0.77 (0.25) | 0.91 (0.67, 0.97) |
| Sorafenib | 25 | 0.70 (0.28) | 0.79 (0.33, 0.94) |
| Other^a^ | * | 0.92 (0.06) | 0.93 (0.89, 0.94) |
| SD, standard deviation; IQR, interquartile range.  * indicates cell value is ≤11 and is suppressed to protect patients’ confidentiality.  ^a^Other includes Axitinib and Everolimus. | | | |

| **Supplemental Table 2.** Proportion of days covered^a^ among patients with metastatic RCC who survived at least 90 days post-OAA initiation (n=207) | | | | | |  |
| --- | --- | --- | --- | --- | --- | --- |
|  | Mean PDC | | Diff. in PDC (95%CL) | | P |  |
| **Patient characteristics** |  |  | |  | | |
| Age at diagnosis |  |  | |  | | |
| 18-49 | 0.83 | Ref | |  | | |
| 50-64 | 0.76 | -0.07 (-0.19, 0.04) | | 0.22 | | |
| 65-69 | 0.77 | -0.06 (-0.17, 0.06) | | 0.34 | | |
| 70-74 | 0.83 | 0.00 (-0.12, 0.13) | | 0.96 | | |
| 70+ | 0.80 | -0.02 (-0.13, 0.08) | | 0.66 | | |
| Patient location |  |  | |  | | |
| Urban | 0.81 | Ref | | - | | |
| Rural | 0.76 | -0.04 (-0.11, 0.02) | | 0.20 | | |
| Insurance at diagnosis |  |  | |  | | |
| Private | 0.90 | Ref | | - | | |
| Any Medicaid | **0.75** | **-0.15 (-0.26, -0.05)** | | **0.004** | | |
| Medicare only | **0.74** | **-0.16 (-0.24, -0.09)** | | **<0.001** | | |
| Number of comorbid conditions |  |  | |  | | |
| 0 | 0.79 | Ref | | - | | |
| 1 | 0.74 | -0.05 (-0.13, 0.03) | | 0.20 | | |
| 2 | 0.84 | 0.04 (-0.04, 0.13) | | 0.30 | | |
| 3 | 0.81 | 0.02 (-0.06, 0.10) | | 0.67 | | |
| Distance to nearby NCI^h^ (miles) |  |  | |  | | |
| Quartiles 1 – 3 | 0.79 | Ref | | - | | |
| Quartile 4 | 0.78 | -0.01 (-0.08, 0.06) | | 0.73 | | |
| Years in practice^d^ | - | 0.00 (-0.00, 0.00) | | 0.36 | | |
| Volume^e^ | **-** | 0.00 (0.00, 0.01) | | 0.33 | | |
| Location |  |  | |  | | |
| Urban Only | 0.81 | -0.06 (-0.13, 0.01) | | 0.10 | | |
| Rural and Urban | 0.75 | Ref | | - | | |
| OAA, oral anti-cancer agent; RCC, renal cell carcinoma.  Bolded estimates have P-values less than 0.05. Variables included in model selected using LASSO approach.  ^a^ Proportion of days covered is out of a total of 90 days.  ^b^Due to small cell sizes, categories of urology/urological surgery, internal medicine, and other were collapsed into one category  ^c^Due to small cell sizes, categories of Non-Hispanic Black, Asian/Pacific Islander, and other were collapsed into one category.  ^d^Years in practice was scaled to 5 years.  ^e^ Provider volume was scaled to 4 RCC patients and covers the period between the patient’s metastatic index date and all prior years of data. | | | | | | |
